# Mr. Chalmers, on Pulmonic Inflammation

**Published:** 1806-12

**Authors:** W. Chalmers

**Affiliations:** Shorncliff Camp


### Medical and Phyfical Journal.

VOL. XVI.] DECEMBER, 1806. [NO. 94.

*Printed for R. PHILLIPS, by W. Thorne, Red Lion Court, Fleet Street, London.*

### *To the Editors of the Medical and Phyfical Journal.*

GENTLEMEN,

AFTER what has been written on pulmonic inflammation, it may appear superfluous in me, to offer any remarks on that subject: I am nevertheless of opinion, that it is a disease of which too great a proportion of patients die; and this, I conceive to be owing, partly, to the affection gaining ground before the symptoms sufficiently evince themselves, or, at least, are noticed; and partly to the forbearance and timidity of the practitioner.

To urge the necessity, therefore, of being more circumspect in the diagnosis, and by far more rigorous in the treatment of pneumonia, than is generally, at present, the practice, is the design of the following observations.

I have seen this disease so fallacious in its attack, as to require great discrimination in discerning it: yea, I have often seen it mistaken for typhus. In those cases it was generally preceded a day or two, by impaired appetite, slight head-ach, wandering pains, and a degree of anxiety. During this period the tongue was generally white, not very dry, but its taste somewhat vitiated: but in a few instances the tongue was foul, covered with a blackish crust, very dry, and furrowed.

The bowels for the most part were costive; sometimes natural; very rarely loose. The skin, although dry, varied little in heat from the human standard; and the pulse, though most frequently above 80, was often below 70.

When in this stage of the disease, emetics were prescribed, they invariably did injury.

The symptoms I have described, were followed with loss of appetite, rigors, head-ach, pain in the loins, great lassitude, prostration of strength to no small degree, anxiety, and oppression about the præcordia; and that too with nausea, and vomiting: the pulse was feeble and small; the patient had no cough, and although a little difficulty of breathing attended, there was no *pain* in the thorax, nor heat much augmented. In a few hours, however, the difficulty of breathing became greater, with the heat of the body much increased; pulse exceeding 90 strokes in a minute, but still buried and small; great thirst and restlessness; hurried respiration, and some degree of pain in the chest, though not confined to any particular spot, nor very acute. If the patient was then bled, the pulse rose, became harder and quicker, and the difficulty of breathing was a little relieved, while at the same time the pain in the thorax grew *more acute* and settled.

Unless the bleeding was carried to a considerable length, the patient soon described the stitch in his breast, as a knife cutting him on a full inspiration. This was followed by a short, dry cough, occasioning much pain.

In a few instances I have known the cough antecedent to every other symptom I have mentioned: It was sometimes attended with a mucous expectoration; at other times it was dry: and I have seen blood coughed up, and mucus streaked with blood, several days *before any pyrexial affection;* I have also known those symptoms disappear, about the time the patient was seized with pneumonia.

The cases of pulmonic inflammation, whose attack seemed secondary to the fever (though the fever I believe to have been originally symptomatic of inflammation going on in the lungs) more frequently occurred in summer: and in every case where immediate and rigorous steps were not taken, the patient either died before the fifth day, in a state of delirium and suffocation; or if, by a few ill-judged bleedings, the disease was protracted, he lingered three or four weeks in agony and distress.

Yet after every hope of recovery had expired, I have known patients become convalescent in the third week, and in less than two months have the whole process of suppuration over. In those cases I never saw an abscess point externally.

The attack of pneumonia, however, is often instantaneous. I have known sailors and soldiers seized in the night in their crowded apartments, with a most severe pain in the thorax, and all the symptoms of pulmonic inflammation: And that too so suddenly, that immediate death must have been the consequence, had there been no medical assistance at hand. Sometimes the pain was so acute acute as to occasion syncope. In those instances one full bleeding, with a warm pure air, generally effected the cure. But if the bleeding was not copious, the disease proved tedious, and its fatality not always prevented by a recurrence to the lancet.

In other cases, a sudden check at the surface, from going warm into a cold atmosphere, or from plunging into the water in a state of perspiration, has brought on inflammation of the lungs; but the disease thus produced I have always found the least dangerous.

I need not notice the common attacks of this disease, nor its combination with hepatitis, or catarrh: nor shall I mention other diseases to which it is somewhat similar in appearance, nor those which it often succeeds: the length of this paper will not permit me.

That inflammation of the lungs is a disease more frequently occurring in cold seasons, I have no hesitation in admitting; but that it often happens in warm seasons I must in turn assert. Although I have observed it in latitudes as high as 70 and 80 north, and also within the tropics, I believe it to be chiefly confined to temperate climates. And I have marked its attack follow sleeping in crowded places, oftener than either checked perspiration, or cold.

As to the predisposing cause of pneumonia, I think whatever renders the body irritable; or tends to incrassate the sanguine fluid, or to impregnate it with acrimony, or to alter the natural cohesion or proportion of its particles; or whatever takes from the lungs their relative tone, may be placed under that head. And as to the exciting cause, whatever destroys the due balance of the circulation, or, prevents its free motion through the lungs, or produces irritation in them, by læsion, distention, or otherwise, seems to me to deserve that name.

The proximate cause of pneumonia, like every inflammation, I conceive to be obstruction; and that produced, by blood being determined to the lungs, either in a greater quantity than it is compatible with their function to receive; or in a state too cohesive to pass, or too acrimonious not to injure their texture. Whether this inability of the blood to pass through the lungs depends oftener on an undue quantity; or on a quality more viscid than natural; on a deficiency of serum; or arises from a want of due assimilation, or from a contraction of the pulmonary vessels, I will not determine. I think, however, there are cases depending severally on each of those causes.

By whatever means obstruction is produced, the heart is exerted in order to overcome it: to all parts of the body this influence of the heart is extended, in a proportion depending on the quantity of stimulus: on the freedom of its motion, and the extent of resistance.

Where the stimulus is sufficient, and the freedom of the motion of the heart uninterrupted, the circulation in every vessel becomes stronger and more rapid. This increase of action produces augmentation of heat, and heat increased keeps up new stimuli.—In the obstructed vessels the force and friction of the circulation being peculiarly felt, and that to a greater degree than is consistent with health, great heat, pain and redness ensue, which continuing, till the force and velocity of the circulation is diminished by some means or other, natural or artificial, keeps up a course of irritation, and produces symptomatic fever.

If in pneumonia, the obstruction is great and extensive, the return of blood from the pulmonary veins will be relatively less than natural; at the *right ventricle* of the heart there will be a partial stagnation, and the return of blood from the venæ cavæ will be interrupted. This interruption will influence the vessels at the surface, by impeding their circulation. Thus we account for the feebleness of pulse I have described; and, as the heart, oppressed with an accumulation of blood, labours in vain to force it through vessels already loaded, it imparts that sensation of buried pulse.

On the other hand, a sudden check at the surface of a person in health, will cause a stagnation at the *left ventricle* of the heart, and as the blood from the lungs is thereby prevented from returning freely, an accumulation will take place in them.

It appears rational to conclude, that the obstruction producing pulmonic inflammation, does not always depend on the same exciting cause, when in cases of equal appearance, the blood taken, exhibits every variety in the proportion of its parts. In some cases, on the first day of the disease, I have seen the coagulum leave so little serum, that in twenty ounces of blood, eighteen became a hard tough cake. On the same day, in another patient, there were ten ounces of serum to eight of coagulum. The serum in one case was colourless, in another greenish, and in a third inclining to white. Its taste varied as much as its appearance. The degree of cohesion in the cake was sometimes very great; at other times extremely small. The coagulable quality was more or less in different patients.

tients. The specific gravity of the serum was uncertain, and its chemical qualities as various.

I allow that some of these phenomena may be accounted for, by the manner of evacuating the blood; by the degree of exertion in the heart, displayed in its effects of augmenting the natural heat; by the quantity of fluid lately received into the stomach of the patient; his habit; and the temperature of the atmosphere, together with its purity, where the blood cooled; but I am as prompt in declaring, that in several cases, where the circumstances nearly coincided, in every respect, except in what I conceived to be the exciting cause, the blood exhibited the appearances and properties I have enumerated.

From what I have advanced with regard to the causes of pneumonia, it follows, that to remove, or at least diminish stagnation when present; or to reduce the force of the heart, when the obstruction is less than to occasion accumulation at either of its ventricles, will be the first step towards a cure. This can only be effected by taking from the general mass of blood, a quantity proportioned to the exigency of the case, and the patient's habit. Venesection therefore ought to take place *immediately* on the slightest appearace of this disease; and, although I do not conceive it to be a contagious one, yet it is often epidemic in some degree, and therefore should be anticipated, and steps taken, which in other cases might seem premature and dangerous. By waiting for a hard pulse, the patient may be lost; and if a repetition of phlebotomy depends on the inflammatory crust of the first bleeding it will seldom take place. I would therefore advise twenty ounces to be taken away when the smallest oppression at the breast is felt, if it is attended with the most distant symptom of fever. An emollient laxative glyster is then to be given. Notwithstanding the happy effects of emetics in the beginning of febrile diseases in general, every evacuant of that class stronger than chamomile tea is to be avoided here, let the affection of the stomach be what it will. A neutral purgative ought to be given as soon as possible, and tepid diluent drinks, mildly demulcent, and acidulated, should be largely administered. At the expiration of *two hours* from the first bleeding, if *any* affection remains, recur to the lancet, and take away a pound: The patient should then be put in the warm bath. Blisters, although in some cases serviceable, are often applied injudiciously. When on the first attack, the pain is fixed to a particular part, attended with cough and much diffi-

 culty culty of breathing, a large blister may be of use. In this case it is unnecessary to wait for any number of bleedings; it should be laid on at first. In general, however, the warm bath supersedes the necessity of blistering, and partial fomentations give also great relief. A blister, if allowed to remain on so long that absorption of flies takes place, or if a great discharge of serum is induced by it, does injury. And as these are too often the effects of blisters, I think they require caution. It need not be remarked that the patient's apartments should be heated to about 80° of Farenheit; and the air thus warmed, as pure as possible. Leeches applied as near the part affected, as conveniently may be, will of course have no bad effect. When other two hours have elapsed, should the smallest affection of chest remain, it will be necessary to take eight or ten ounces of blood, and this repeated *every two hours*, till no symptom of dyspnœa is left. In no one instance did I ever see copious bleeding unsuccessful; while on the other, I have seen many suffer through a timid restriction. Another enema ought to be given six or eight hours after the first, and again repeated at the same interval. If much head-ach attends, treat it with cold; pediluvium is also indicated. As to the exhibition of nourishment, something farinaceous but not heating, may be conveyed into the stomach in the patient's drink; and as to medicines, few will be necessary. If any saline draughts are prescribed, the prepared kali, with lemon juice, sufficiently diluted, is among the best.

It is unnecessary to advert to the necessity of having the patient confined to bed, even in slight attacks of this complaint, and of his refraining from speaking: a darkened apartment is always to be preferred.

Such are the steps to be taken when we find the disease in its infancy; but it often happens that a day or more elapses from its commencement to the time of calling in medical assistance: even then I conceive the same treatment to be proper, and as far as the fourth or fifth day. I have bled with success in cases less rapid in their progress on the sixth and seventh day of the disease.

As to the exhibition of nitre and pectoral medicines in pneumonia, I have seldom observed much good accrue from them, even where the cough was troublesome, especially in the first instance.

Opium, where the phlogistic diathesis is taken off, may be useful; and, on the second and third day, opium combined with camphor, and a small proportion of calomel, is is given with happy effects, especially where the determination to the skin seems still to linger.

Of sweating, in the beginning of this disease, I disapprove. A gentle diaphoresis may be excited by diluent drinks and neutrals, but more particularly when checked perspiration brought on the malady.

Under the above treatment, pneumonia will seldom exist many hours: if the plan therefore calls forth a more vigorous practice than is generally adopted in similar cases, I have no hesitation in asserting that the proportion of victims to pneumonia will be considerably diminished.

I am, &c.

*Shorncliff Camp,*

*September* 8, 1806.

W. CHALMERS.

